# Dataset for file fragment classification of video file formats

**DOI:** 10.1186/s13104-020-05037-x

**Published:** 2020-04-15

**Authors:** Narges Sadeghi, Mohadeseh Fahiminia, Mehdi Teimouri

**Affiliations:** Information Theory and Coding Laboratory, University of Tehran, Tehran, Iran

**Keywords:** Video file formats, Classification, File formats, File fragments

## Abstract

**Objectives:**

File fragment classification of video file formats is a topic of interest in network forensics. There are some publicly available datasets for file fragments of various file types such as textual, audio, and image file formats. However, there is no public dataset for file fragments of video file formats. So, in order to evaluate and compare the performance of the classification methods, a challenge is the need to have such datasets.

**Data description:**

In this study, we present a dataset that contains file fragments of 10 video file formats: 3GP, AVI, ASF, FLV, MKV, MOV, MP4, WebM, OGV, and RMVB. Corresponding to each format, the dataset contains the file fragments of video files with different video codec types: H.263, MPEG-4, WMV, H.264, FLV1, H.265, VP8, VP9, Theora, and RealVideo. Totally, 20 different pairs of video format and codec are employed. For each pair of video format and codec, 30,000 file fragments are provided. Totally, the dataset contains 600,000 file fragments.

## Objective

A huge part of the Internet traffic is used for transmitting video files. Due to the large size of these files, they are segmented into fragments. The fragments are communicated over the network. Some of these fragments can be received by the network surveillance unit. The network surveillance unit may desire to identify the file format of each fragment for network forensics purposes.

Some researches have been carried in the field of file fragment classification of video file formats [1–3]. There are some publicly available datasets for file fragments of various file formats [4–6]. However, there is no public dataset for file fragments of video file formats. This makes it difficult for other researchers to compare the proposed methods with the existing methods.

In this study, we present a dataset that contains file fragments of 10 video file formats: 3GPP file format (3GP), Audio Video Interleaved (AVI), Advanced systems format (ASF), Flash Video (FLV), Matroska Video (MKV), QuickTime Movie (MOV), MPEG-4 Part 14 (MP4), Web Movie (WebM), OGV, and RealMedia Variable Bitrate (RMVB). Corresponding to each format, the dataset contains the file fragments of video files with different video codec types: H.263, MPEG-4, Windows Media Video (WMV), H.264, FLV1, H.265, VP8, VP9, Theora, and RealVideo.

## Data description

First, the whole set of the original video files are prepared. These files are taken from a private archive of Iranian movies and television series. The format of these files is either MKV or MP4. All these videos are encoded by H.264 codec. The frame rate of these videos varies from 15 to 25 frames per second with a frame size from 384 × 288 to 1920 × 1090. The audio codec of the original video files is either Advanced Audio Coding (AAC) or MPEG Audio Layer-3 (MP3) with bitrates from 128 to 320 kbps.

The set of the original video files are converted in order to obtain video files in 20 different pairs of format/codec: 3GP/H.263, 3GP/MPEG4, AVI/WMV, AVI/H.264, AVI/MPEG4, ASF/WMV, FLV/FLV1, MKV/H.264, MKV/H.265, MKV/MPEG4, MOV/H.264, MOV/H.265, MOV/MPEG4, MP4/H.264, MP4/H.265, MP4/MPEG4, WebM/VP8, WebM/VP9, OGV/Theora, and RMVB/RealVideo.

Regardless of video format/codec, the contents of any two converted video files are not the same. For each pair of format/codec, we have 60 compressed videos. So, totally we have 1200 video files. Each of these files is segmented into 1 Kbyte (i.e. 1024 bytes) fragments. Then, 500 fragments are randomly selected among the fragments of each file. Same as [6], before randomly selecting the fragments, 12.5% of the initial fragments and 12.5% of the final fragments of each file are discarded.

For each pair of video format/codec, we have 30,000 file fragments. So, the dataset of file fragments contains 600,000 file fragments. The dataset is partitioned according to 20 different pairs of video format/codec. Each partition is represented by an individual data file shown in Table 1. For example, data file 1 (i.e. 01. 3gp–H.263.dat) contains 30,000 fragments of 3GP/H.263 files. Data files are provided in a generic binary data file format with.dat file extension.

**Table 1:** Overview of data files/data files

Label	Name of data file/data file	File types (file extension)	Data repository (DOI)
Data file 1	01. 3gp–H.263	Generic binary data (.dat)	OSF (10.17605/OSF.IO/8HM5S)
Data file 2	02. 3gp–MPEG4	Generic binary data (.dat)	OSF (10.17605/OSF.IO/8HM5S)
Data file 3	03. AVI–WMV	Generic binary data (.dat)	OSF (10.17605/OSF.IO/8HM5S)
Data file 4	04. AVI–H.264	Generic binary data (.dat)	OSF (10.17605/OSF.IO/8HM5S)
Data file 5	05. AVI - MPEG4	Generic binary data (.dat)	OSF (10.17605/OSF.IO/8HM5S)
Data file 6	06. ASF–WMV	Generic binary data (.dat)	OSF (10.17605/OSF.IO/8HM5S)
Data file 7	07. FLV–FLV1	Generic binary data (.dat)	OSF (10.17605/OSF.IO/8HM5S)
Data file 8	08. MKV–H.264	Generic binary data (.dat)	OSF (10.17605/OSF.IO/8HM5S)
Data file 9	09. MKV–H.265	Generic binary data (.dat)	OSF (10.17605/OSF.IO/8HM5S)
Data file 10	10. MKV–MPEG4	Generic binary data (.dat)	OSF (10.17605/OSF.IO/8HM5S)
Data file 11	11. MOV–H.264	Generic binary data (.dat)	OSF (10.17605/OSF.IO/8HM5S)
Data file 12	12. MOV–H.265	Generic binary data (.dat)	OSF (10.17605/OSF.IO/8HM5S)
Data file 13	13. MOV–MPEG4	Generic binary data (.dat)	OSF (10.17605/OSF.IO/8HM5S)
Data file 14	14. MP4–H.264	Generic binary data (.dat)	OSF (10.17605/OSF.IO/8HM5S)
Data file 15	15. MP4–H.265	Generic binary data (.dat)	OSF (10.17605/OSF.IO/8HM5S)
Data file 16	16. MP4–MPEG4	Generic binary data (.dat)	OSF (10.17605/OSF.IO/8HM5S)
Data file 17	17. WebM–VP8	Generic binary data (.dat)	OSF (10.17605/OSF.IO/8HM5S)
Data file 18	18. WebM–VP9	Generic binary data (.dat)	OSF (10.17605/OSF.IO/8HM5S)
Data file 19	19. OGV–Theora	Generic binary data (.dat)	OSF (10.17605/OSF.IO/8HM5S)
Data file 20	20. RMVB–RealVideo9-10	Generic binary data (.dat)	OSF (10.17605/OSF.IO/8HM5S)
Data file 21	SettingsTable	Portable document format (.pdf)	OSF (10.17605/OSF.IO/8HM5S)
Data file 22	ConversionSettings	Archive file format (.zip) containing 20 portable network graphics (.png) files	OSF (10.17605/OSF.IO/8HM5S)
Data file 23	ReadFragments	Matlab script file (.m)	OSF (10.17605/OSF.IO/8HM5S)

Data file 21 (i.e. SettingsTable.pdf) contains a table, in which, the software program employed for generating the videos of each pair of format/codec is specified. In this table, the employed compression settings are also specified. Data file 22 (i.e. ConversionSettings.zip) contains several screenshots of the software programs that display the employed compression settings. These screenshots actually demonstrate how the videos are converted. Data file 23 (i.e. ReadFragments.m) is a script in MATLAB language that reads all the fragments from one or more data files. The details of this script are given in [6].

## Limitations

- The size of the fragments is considered to be fixed and equal to 1024 bytes.
- A defined subset of video formats and codecs are considered.
- The number of raw data files for each pair of video format and codec is 60.

## Data Availability

The data described in this Data note can be freely and openly accessed on OSF at 10.17605/OSF.IO/8HM5S [7]. Please see Table 1 and reference list for details and links to the data.

## Abbreviations

3GP: 3GPP file format
AAC: Advanced Audio Coding
ASF: Advanced systems format
AVI: Audio Video Interleaved
FLV: Flash Video
MKV: Matroska Video
MOV: QuickTime Movie
MP3: MPEG Audio Layer-3
MP4: MPEG-4 Part 14
RMVB: RealMedia Variable Bitrate
WebM: Web Movie
WMV: Windows Media Video
